# Neural activation patterns in open-skilled and closed-skilled athletes during motor response tasks: insights from ERP analysis

**DOI:** 10.3389/fspor.2024.1476210

**Published:** 2024-11-19

**Authors:** Viktors Veliks, Dinass Talents, Andra Fernate, Klavs Evelis, Aleksandrs Kolesovs

**Affiliations:** ^1^ RSU Latvian Academy of Sport Education, Rīga Stradiņš University, Riga, Latvia; ^2^ Faculty of Medicine and Life Sciences, University of Latvia, Riga, Latvia

**Keywords:** open and closed-skill sports, Choice Response Time task, lead and trailing hands, ERP, neuronal control

## Abstract

The present study explored behavioral outcomes and neural correlates of cognitive control abilities in open-skill sports athletes compared with closed-skill sports athletes. The participants of the study were 16 right-handed male athletes. Nine basketball players formed a group of athletes from open-skill sports, and seven outdoor track and field runners formed a comparison group for closed-skill sports. During the two-color Choice Response Time task with simultaneous EEG registration, psychophysiological observation was performed to assess athletes’ functioning. A significant interaction between a sports type and the hand reveals more symmetrical functioning of the hands in basketball players, which is also confirmed by the neural activity of brain regions responsible for motor action (C3 and C4). Although there was no main effect of the sport type, the study revealed closer patterns of motor action and neural regulation of the left and right hand in open-skilled athletes than in closed-skilled athletes.

## 1 Introduction

Sports can be categorized based on many characteristics involving intensity, duration, movement dynamics, type and skill, isometric and isotonic components of exercise, team and individual sports, and other bases for classification (1, 2). However, when interested in the research of respective sports’ cognitive demands and, therefore, its effects on practitioners’ executive functions, predominant classification is based on respective sports’ environmental and task requirements—resulting in the classification of open and closed-skill sports (3–5). Nonetheless, this division is not dichotomous and should be considered a continuum (6).

Sports with external conditions influencing the choice of action are at one end of the continuum. They are called open-skill sports and include basketball, football, baseball, martial arts, boxing, volleyball, and others (7, 8). Sports with relatively constant and predictable external conditions and relatively independent self-paced action are at the other end of the continuum (7). They are called closed-skill sports, such as athletics, swimming, or artistic gymnastics. It is worth noting that few open-skill sports demand the use of both upper and lower extremities more or less equally and have been regarded as the furthest on the open and closed-skill spectrum (Category 4) due to countless unpredictable dynamic environmental factors (7, 9).

One such sport is basketball (7, 9), which is a technically and tactically complex sport characterized by a high-intensity load with a change in the direction and dynamics of movements every 1–2 s (10). During the game, players must constantly evaluate and adapt their actions to rapidly changing external conditions—the position of opponents and teammates, the dynamics of their movement, the direction of the ball's movement, speed, distance, trajectory, and many other factors. Because of rapidly changing external factors, athletes participating in open-skill sports need highly developed cognitive abilities, including inhibitory control, which is one of the executive functions defined as the ability to control attention, thoughts, and behavior to suppress an automatic response and instead choose the most appropriate one for the given situation (11). For example, during a basketball game, a player sees an open teammate in an advantageous position under the basket. However, before the pass is made, the defender rushes in front, and the pass is no longer possible. To avoid a mistake, the player must be able to instantly inhibit the initial reaction by choosing another more relevant to the situation, for example, making a basket.

In closed-skill sports, the external environment is much more fixed, and the athlete knows upcoming events and can prepare for them in advance, which is not valid for open-skills sports since there are endless situations and game plan varies from game to game. For example, in one of many closed-skill sports—javelin throw, there is more or less one complex movement mastered that can be regarded as a “sport itself.” In addition, the length and direction of the run are fixed. There are no direct opponents in front trying to interfere and make unpredictable actions, and there are complex rules to obey. Since there is no necessity to react and adapt rapidly to highly paced, everchanging occurrences, inhibitory control is much less decisive in such sports, and athletes’ performance depends on physical, technical, and tactical abilities (12).

Studies on athletes’ inhibitory control (13–15) do not directly compare basketball players, representing the most challenging Category 4, with closed-skill athletes. There also is a limited number of studies in other open-skill sports showing superior inhibitory control abilities compared to athletes participating in closed-skill sports: tennis vs. swimmers (16, 17); baseball vs. track and field and swimmers (18); mixed open vs. closed-skill sports senior athletes (19). The lack of research regarding inhibitory control analysis in open vs. closed-skilled athletes can also be identified in a recent systematic review (20). Only nine studies compared these groups, and no publications included highly paced team sports such as basketball as an open-skilled group.

The importance of further research is also supported by the negative findings of a meta-analytical study (7), presenting no significant effect of open vs. closed-skill sports on executive functions, including inhibitory control. In addition, there is a continuous discussion on differences and controllable factors for athletes of different sports and age groups (8, 21). Therefore, it is necessary to continue exploration of the specific effects of open-skill sports on athletes’ cognitive functioning.

Combining behavioral and neurobiological markers of athletes’ functioning provides broader opportunities for specifying the effects of the sports type (22, 23). Functional magnetic resonance imaging (fMRI) indicated that open-skill sports can improve athletes’ inhibitory control when both types of sports improve athletes’ reaction time compared with the sedentary group (22). In turn, electroencephalography (EEG) demonstrated the effects of sports on inhibitory control, forming a resource for maintaining executive functions during aging (23). However, the EEG study (23) did not involve lateral regions of interest associated with executive control (frontal) and sensorimotor reactions (central).

We have considered EEG the most appropriate method for the present study. Compared to fMRI, EEG has a high temporal resolution at milliseconds [e.g., (24)]. In addition, fMRI uses the blood oxygenation level, which describes correlates of neural activity rather than direct activity. Moreover, using EEG can add neural correlates to the effects of motor performance of basketball players (25), indicating possible differences in the processing of stimuli [e.g., (26)] and post-response functioning [e.g., (27)]. Therefore, the present study aimed to study behavioral outcomes and neural correlates of inhibitory control abilities in open-skilled athletes compared with closed-skilled athletes.

## 2 Method

### 2.1 Participants

For this exploratory study, we assessed the sample size using the GPower 3.1 program (28) with an alpha level of 0.05 and power of 0.80 for F-tests with two groups (sports type), two repeated measures (left and right hands), and correlation between measures of 0.48 (29). The sample size was at a minimum of *n* = 16 for detecting large effects (*η*^2^ ≥ 0.14).

The participants of the study were 16 right-handed male athletes. Nine basketball players formed a group of athletes from open-skill sports (21.0 ± 1.9 years; 192.1 ± 6.4 cm; 92.1 ± 13.8 kg; and BMI 24.9 ± 2.6). Seven outdoor track and field runners formed a comparison group of closed-skill sports (24.1 ± 2.4 years; 183.1 ± 4.6 cm; 79.7 ± 4.6 kg; and BMI 21.8 ± 1.7). The inclusion criteria for both groups were male, age 18–29, being a competitive non-professional athlete, and voluntary participation. The exclusion criteria were color blindness and an upper extremity or back injury at the time of the experiment.

### 2.2 Measures

**Figure 1:**
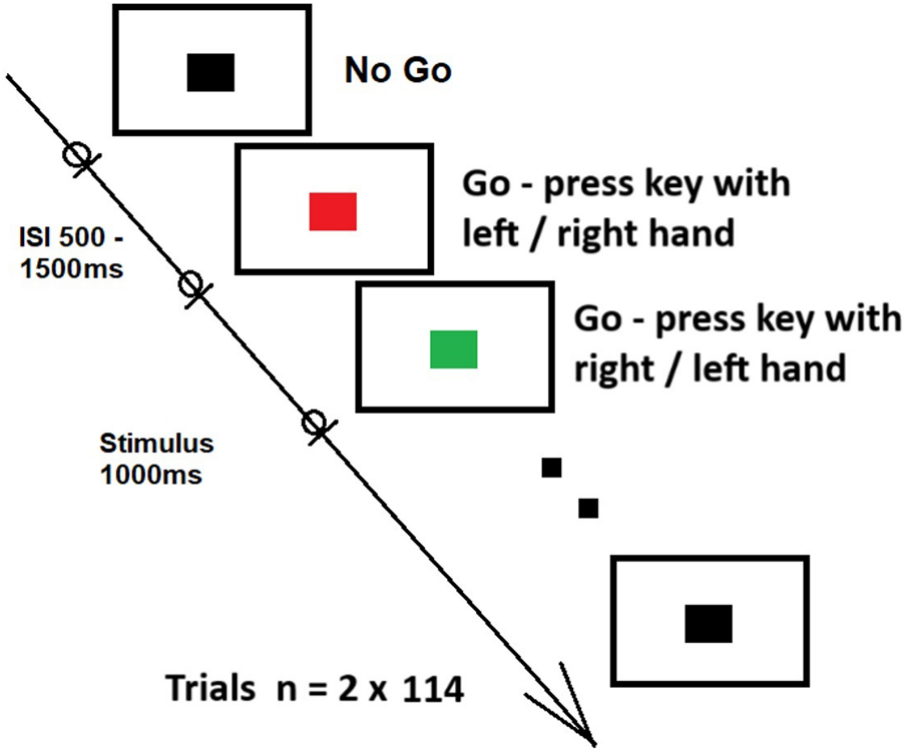
Schematic representation of the choice response time task.

For the psychophysiological assessment, we applied the standard two-color Choice Response Time task (CRT) with simultaneous EEG registration (30). Using Matlab-based (The MathWorks, Inc) PSYCHOTOOLBOX (http://psychtoolbox.org) custom-coded scripts of the CRT task, two color stimuli (red and green) for reacting by the right and left hand and a discriminative stimulus (black) for non-reacting were presented on an LCD screen (see Figure 1). Stimulus and response events were synchronized with EEG amplifier NVX-136 (NVX136, Medical Computer Systems Ltd.) using the application software. The experiment contained four rounds. Each round consisted of 114 trials (456 trials in total). After the round, participants switched the response keys. For the red stimulus, participants pressed the “Z” key during the first and third rounds and the “2” key (on a numeric keypad of the standard keyboard) in the second and fourth rounds. For the green stimulus, the “2” key was used in the first and third rounds, while the “Z” key was used in the second and fourth rounds. Between rounds, the test instructions were displayed on the screen for 60 s between the rounds. The participants completed the task alone while sitting in an armchair in an isolated dark room 1.5 meters from the stimulus-presenting monitor.

EEG recording was conducted with a sample rate of 1,000 Hz and cut-off frequencies of 0.1–100 Hz with impedances < 5 kΩ, which included the 32 standard electrodes of the 10–10 system. A1 and A2 electrodes were reference channels. Acquired EEG recordings were preprocessed and analyzed using Matlab-based (version 2020a, The MathWorks, Inc) EEG analysis software product, EEGLAB (http://sccn.ucsd.edu/eeglab), applying some custom data processing scripts. The EEG was re-referenced to the computed average reference. 50 Hz noise in EEG signals was removed using 48–52 Hz bandpass filters. Then, EEG signals with performance errors or remaining artifacts exceeding ± 100 μV in any channel and eye-blinking artifacts were removed from data using the ICA procedure [based on the online EEGLAB tutorial (31)] before the processing. Additionally, EEG trials were inspected visually before calculating the event-related potentials (ERP) using the ERPLAB plugin (https://erpinfo.org/erplab).

The following electrodes were selected for region of interest (ROI): frontal cortex regions (F7, F3, F4, and F8) participated in cognitive control of the response, involving inhibitory control as an executive function (32, 33), and sensorimotor cortex regions (C3 and C4) involved in the execution of the motor behavior [e.g., (33)]. The correct reaction during the color choice (red- or green-colored squares) included motor behavior, while the correct reaction to the discriminative stimulus (black-colored square) considered no motor behavior. The opposite behavioral pattern represented incorrect reactions, which are usually rare events and are in question for ERP analysis in relatively small groups of respondents. EEG data for the ERP analysis were processed using the EEGLAB STUDY pipeline, and behavior responses were processed using IBM SPSS Statistics (version 29, IBM) at a statistical significance threshold of *p* < 0.05.

## 3 Results

### 3.1 Behavioral outcomes

Participants made a relatively small number of mistakes during the tests (262 mistakes vs. 6,386 correct answer events among all participants and trials). This number did not allow us to perform the correct ERP analysis for errors. Therefore, we also skipped incorrect answers in behavioral analysis except for the level of accuracy in athletes’ responses.

Table 1 presents the reaction times and response accuracy in the open-skilled (basketball players) and closed-skilled (track and field runners) groups. The Shapiro-Wilk test demonstrated no significant deviance from the normal distribution of reaction time for the left (*p* = 0.826) and right (*p* = 0.110) hands in the open-skilled group, and the left (*p* = 0.095) and right (*p* = 0.071) hand in the closed-skilled group. In addition, Box's test confirmed the equality of covariance matrices of the reaction time across groups, Box's M = 2.87, F (3; 21,416) = 0.83, *p* = 0.492. Similarly, there was no significant shift from the equality of covariance matrices of the accuracy of the response, Box's M = 2.89, F (3; 21,416) = 0.81, *p* = 0.488. The Shapiro-Wilk test also demonstrated no significant deviance from the normal distribution of accuracy for the left (*p* = 0.336) and right (*p* = 0.810) hands in the open-skilled group and the right hand (*p* = 0.394) in the closed-skilled group. The accuracy of the left hand in the closed-skilled group had some deviation from the normal distribution (*p* = 0.002). In general, these tests indicated the applicability of mixed 2 (Group) × 2 (Hand) ANOVA for a comparison of reaction time and accuracy of the response.

**Table 1:** Descriptive statistics and mixed ANOVA on reaction time and response accuracy by sport type and hand for the “Go” condition.

Hand	Open-skill sports (*n* = 9)		Closed-skill sports (*n* = 7)		Total (*N* = 16)	
	RT *M* ± *SD*, ms	Accuracy *M* *±* *SD,%*	RT *M* ± *SD*, ms	Accuracy *M* *±* *SD,%*	RT *M* ± *SD*, ms	Accuracy *M* *±* *SD,%*
Left	529 ± 80	91.9 ± 5.3	578 ± 94	89.7 ± 8.5	551 ± 87	90.9 ± 6.7
Right	472 ± 58	95.7 ± 2.5	458 ± 60	96.4 ± 2.3	466 ± 57	96.0 ± 2.3
Total	501 ± 67	93.8 ± 3.4	518 ± 71	93.0 ± 4.4	508 ± 67	93.4 ± 3.8
Effects	*F* (1, 14)		*p*		*η* ^2^	
	RT	Accuracy	RT	Accuracy	RT	Accuracy
Group	0.25	0.14	0.624	0.716	0.02	0.01
Hand	44.20	9.25	**<0.001**	**0.009**	0.76	0.40
Group × Hand	5.66	0.68	**0.032**	0.423	0.29	0.05

RT, reaction time. Significant differences are highlighted in bold.

**Figure 2:**
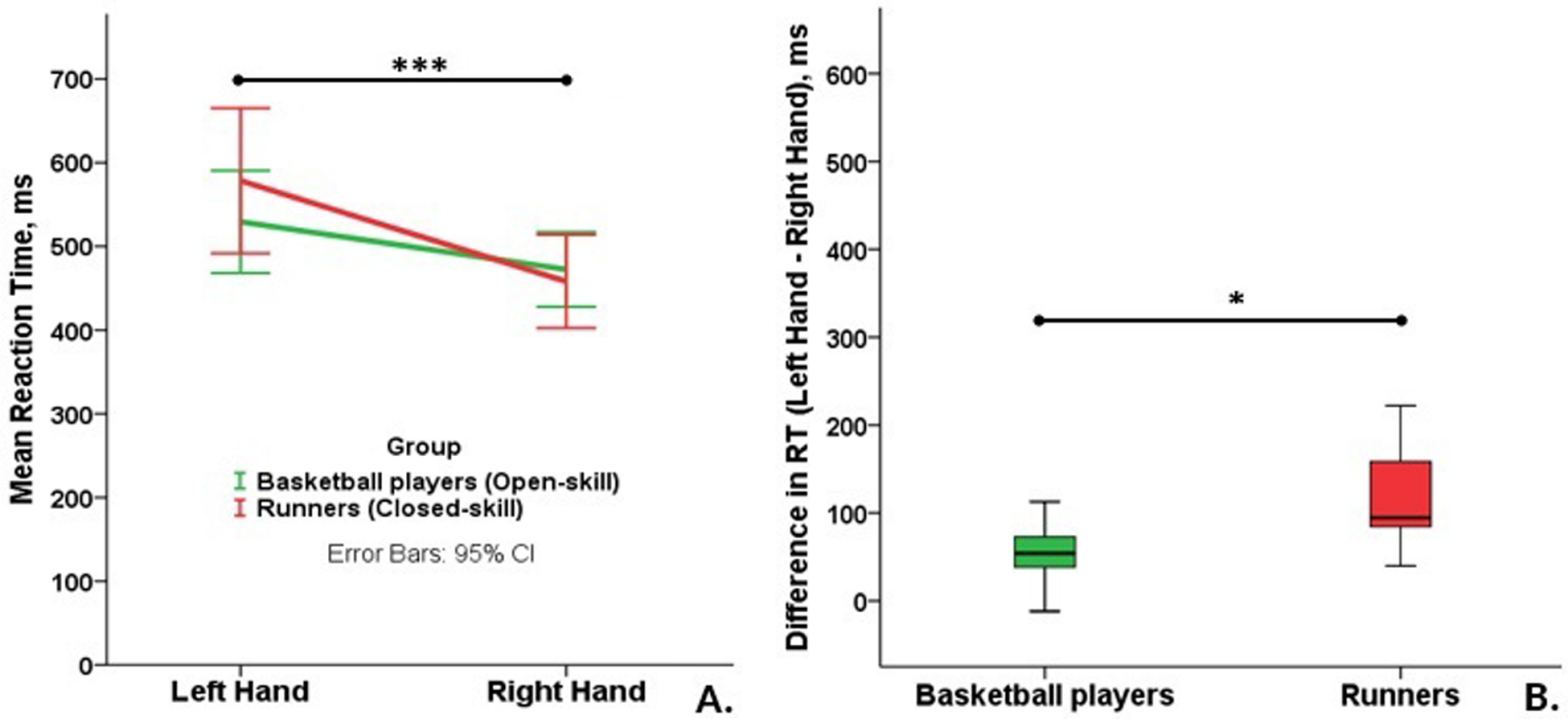
Comparison of **(A)** the reaction time (RT) during correct answers of open- and closed-skilled groups and **(B)** differences between RT of the left and right hand (***p < 0.001, *p < 0.05).

The results (Table 1) revealed no significant main effect of the group of athletes on their reaction time. Simultaneously, the main effect was on the athlete's hand. The reaction time for the left hand was longer than for the right one (Figure 2A). The effect size estimate (*η*^2^) indicated a large effect, accounting for 76% of the variance in the reaction time. The effect size of the significant interaction between the group and hand was also relatively large (29% of variance explained). However, this effect required a deeper exploration of the results. The following comparisons revealed no significant differences between open- or closed-skilled groups in the reaction time on the left, t (14) = −1.13, *p* = 0.276, or the right hand, t (14) = 0.48, *p* = 0.637. The interaction expressed as a significant difference between left and right hands’ reaction time in groups, t (14) = 2.38, *p* = 0.032, Cohen's d = 1.14. It was 120 ± 68 ms in the closed-skilled group and 57 ± 38 ms in the open-skilled group (Figure 2B).

In addition to effects on reaction time, the hand's main effect was significant for the accuracy of the response. The effect size was relatively large (40% of variance explained). It did not interact with the sport type, and the sport type also had no main effect on the response accuracy.

### 3.2 Neural correlates

**Figure 3:**
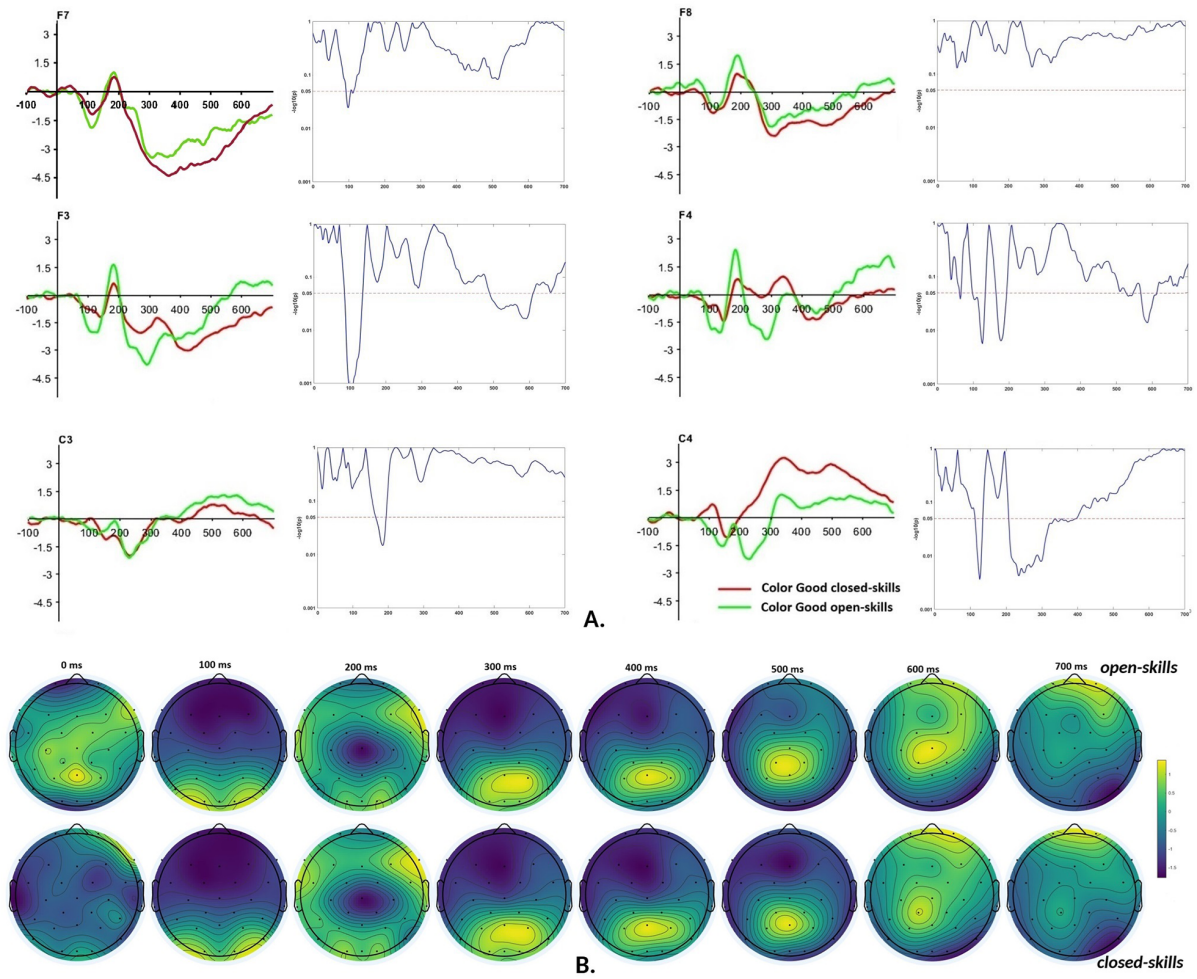
Time-amplitude plots of ERP for correctly recognized stimuli in frontal (ROI F3, F4, F7, and F8) and motor (ROI C3 and C4) cortex in open- and closed-skilled athletes and corresponding *p*-levels of *t*-test **(A)**; topoplot of EEG map of activation patterns for correct responses in the interval from 0 to 700 ms after stimulus onset in open-skills and closed-skills groups **(B)**.

Figure 3A presents the comparison of ERP in ROI selected for the study. Frontal ROI F7 and F8 demonstrated relatively similar ERP trends, except for a relatively small difference between groups at 100 ms in F7. In contrast, less lateralized ROI—F3 and F4—demonstrated more noticeable differences between the athletes from open and closed-skill sports. The significant differences were revealed at 100 ms for both ROIs. In both hemispheres, the signal amplitude was higher in the open-skill group. Simultaneously, a significant difference was revealed in F4 at 200 ms. It indicated a higher amplitude of ERP in the open- than closed-skilled group. Therefore, the neural activity of the right frontal lobe demonstrated more intensive functioning in the open-skilled group than in the closed-skilled group during the stimulus processing. After the response (within 500–600 ms), the F3 activity was more prolonged from a negative spike at 400 ms in the closed-skilled group than in the open-skilled group. At the same time, ERP in F3 and F4 indicated forming a new positive spike in the open-skilled group.

In contrast to frontal regions, ROI C3 indicated slightly lower activation in the open-skilled group than in the closed-skilled group at 200 ms during the two negative spikes before and after 200 ms. In turn, the analysis of C4 activity revealed two negative spikes in the open-skilled group (similar to C3 in both groups) and one negative spike in the closed-skilled group, followed by a positive spike and prolonged high-amplitude activation leading to significant differences from 200 to 400 ms. Simultaneously, ERPs in C3 and C4 of the open-skilled group were closer to each other than those in the closed-skilled group. However, a spike in the interval of 300–350 ms in C4 was observed in both groups, which differed from ERP in C3. Therefore, motor regulation of the left hand before the motor response was associated with higher activation in the closed-skilled than in the open-skilled group.

Figure 3B presents topoplots as an integrated view of brain activity in the groups under investigation. The interval from 100–400 ms demonstrated similarities in the EEG signal averaged amplitude in both groups. Some differences between groups were expressed in frontal and central regions from 500 ms, associated with the motor response. Together with the analysis of ERP, it indicates an association of the sport type with differences in the post-response processes.

## 4 Discussion

The results generally indicate no main effect of the sports type and a significant interaction between a sports type and the hand. The closer behavioral patterns of hand functioning are more evident in open-skill sports than in closed-skill sports, which is also confirmed by the neural activity of brain regions responsible for motor action of the right (C3) and left (C4) hands. Therefore, the results confirm no simple improvement in the open-skill sport compared to the closed-skill sport (7). It also concurs with the results indicating that both types of sports can improve athletes’ reaction time (22).

Simultaneously, behavioral outcomes and their neural correlates show consistent effects of the sport type in the interaction with regulating hands’ functioning. The motor control of the left hand differs from that of the right hand in both groups of sport types. However, the motor control of the left hand in the open-skill group is associated with lower effort and is closer to the control of the right hand than in the closed-skill group. The large effect size for this difference indicates a significant functional shift. This finding points to a need to deal with specific characteristics of the selected open-skill sport because some of them (e.g., fencing) consider a predominant involvement of one hand and individual participation (23), while other open-skill sports (e.g., basketball) have more distributed load on both hands and team participation (10).

Together with differences in motor action and its regulation, the study revealed significant differences in the functioning of frontal regions. At the information processing and analysis stages, the frontal lobes were more engaged in the open than the closed-skill group. In contrast, later time slots indicated higher involvement of less lateralized regions (F3 and F4) in the closed than the open-skill group. Therefore, participants from the open-skill sport demonstrated more intensive perceptual analysis [e.g., (26)] and faster resolving after the correct response (27) than those from the closed-skill sport. It concurs with findings on better hand dexterity in basketball players demonstrating better motor skills than non-athletes and adolescent athletes from other sports (25). For example, basketball players performed better in the handgrip tests for dominant and non-dominant limb scoring.

The main limitations of this exploratory study are a relatively small sample size and an unexpectedly low level of errors during the Choice Response Time task. The small sample size allows us to detect only relatively large effects. However, a larger sample size is required to confirm the behavioral trends revealed in this exploratory study. In turn, the low level of errors limited the calculation of ERP under incorrect answers. Therefore, further research is needed to identify the effects of the sport type on dealing with errors. In addition, the involvement of right-handed participants limits the generalization of the results to left-handed athletes.

In conclusion, the findings indicate that, although the sport type had no main effect, the study found closer results of hands’ motor action in open-skilled athletes than in closed-skilled athletes. Data on neural regulation of the left and right hands in open-skilled athletes supports this trend, showing higher consistency than in closed-skilled athletes.

## Data Availability

The raw data supporting the conclusions of this article will be made available by the authors, without undue reservation.
